# A Field Verification Denoising Method for Partial Discharge Ultrasonic Sensors Based on IPSO-Optimated Multivariate Variational Mode Decomposition Combined with Improved Wavelet Transforms

**DOI:** 10.3390/s25247506

**Published:** 2025-12-10

**Authors:** Tienan Cao, Yufei Cui, Haotian Tan, Wei Lu, Fuzeng Zhang, Kai Liu, Xiaoguo Chen, Yifan Chen, Lujia Wang

**Affiliations:** 1 China Southern Power Grid Electric Power Research Institute Co., Ltd., Guangzhou 510700, China; 2 School of Electrical Engineering, China University of Mining and Technology, Xuzhou 221116, China

**Keywords:** partial discharge ultrasonic sensor, field verification, noise suppression, sensitivity, IPSO, energy entropy

## Abstract

Field verification of contact-type ultrasonic sensors enables rapid evaluation of their sensitivity performance, thereby ensuring the accuracy of partial discharge (PD) ultrasonic monitoring results. However, during the verification process, both the standard sensor and the sensor under testing are inevitably affected by ambient noise when receiving verification signals, which can result in significant errors in the verification outcome. To address this issue, a noise suppression method is proposed in this study, which integrates multivariate variational mode decomposition (MVMD) optimized by an improved particle swarm optimization (IPSO) algorithm with a hyperbolic tangent-modulated exponential decay wavelet thresholding technique. First, the IPSO algorithm is employed to automatically optimize the parameters of MVMD. Then, the dominant components of the verification signal are selected based on the energy entropy of each decomposed mode. Subsequently, a novel wavelet threshold function incorporating hyperbolic tangent modulation and exponential decay is constructed and combined with an improved thresholding strategy to denoise the residual noise in the dominant components. Finally, a verification platform based on a real-type transformer is established. Both simulated and measured signals are denoised and subjected to sensitivity verification using the proposed method. Comparative analysis with noise-affected verification results demonstrates that the proposed method effectively suppresses noise in the verification signals and improves the accuracy of the sensitivity verification.

## 1. Introduction

Partial discharge (PD), as an early manifestation of discharges in transformer oil, can be employed to initiate an “active” defense mechanism prior to main insulation breakdown. By utilizing PD signals to trigger protective actions, preventive measures can be implemented before severe arc faults occur [1,2]. Consequently, higher requirements are imposed on the reliability of PD monitoring data. Owing to their flexible installation and high accuracy, contact-type ultrasonic sensors are widely applied in the field for PD detection and localization in transformers [3]. The reliability of the monitoring data is closely related to sensor performance, among which sensitivity exerts the most significant influence on the acquisition of ultrasonic signals [4]. To ensure the reliability of PD-triggered protection technology, it is therefore essential to conduct on-site verification of sensor sensitivity so that critical performance parameters, such as sensitivity, can be obtained quickly and accurately.

However, the complex noise environment in the field interferes with the signals received by sensors, thereby affecting the verification of their sensitivity. Previous studies on the verification of devices such as current transformers and robotic sensor systems have revealed that random phase noise, vibration noise, and other disturbances can exert adverse effects on verification accuracy [5,6]. Therefore, it is essential to clarify the impact of substation noise on the sensitivity verification of ultrasonic sensors and to suppress such noise during the verification process in order to ensure accuracy.

In recent years, numerous studies have focused on denoising methods for ultrasonic signals. Noise suppression approaches based on signal decomposition have been shown to be effective in extracting original signals, including singular value decomposition (SVD), adaptive filtering algorithms, empirical mode decomposition (EMD), and variational mode decomposition (VMD). Keyvannia et al. [7] exploited the differences between the singular values of noise and those of an original signal to classify them by selecting appropriate thresholds, thereby achieving noise suppression. However, the singular values of white noise and original signals are often interlaced, and the manual determination of thresholds is challenging. Shukla et al. [8] employed the EMD method for adaptive denoising in power quality assessment; nevertheless, issues such as mode mixing remained. To overcome this limitation, ensemble empirical mode decomposition (EEMD) was proposed, which effectively alleviates mode mixing [9,10]. Despite this improvement, the computational burden of EEMD remains considerable. Li et al. [11] successfully applied the variational mode decomposition (VMD) method to effectively extract bearing fault features under complex noise environments. However, the performance of VMD strongly depends on the selection of the penalty factor *α* and the number of decomposition modes *K* [12,13]. If inappropriate optimization algorithms are employed for tuning these preset parameters, not only is the decomposition accuracy affected, but the time efficiency of the method is also reduced. Moreover, considering the non-stationary characteristics of ultrasonic signals acquired during verification, wavelet transform has been shown to provide good noise suppression in the time–frequency domain [14,15]. Nevertheless, the choice of wavelet thresholding criteria and threshold functions must be adjusted according to specific application scenarios.

In summary, to address the limited adaptability of existing denoising methods in complex field environments, as well as the reduced sensitivity calibration accuracy caused by signal distortion from traditional thresholding functions, a calibration noise suppression method based on IPSO-optimized MVMD-EEn and improved wavelet thresholding is proposed. First, the IPSO algorithm is employed to automatically optimize the number of decomposition modes and the penalty factor in MVMD. Second, the dominant components of the verification signal are identified by calculating the energy entropy of each modal component. Finally, an arctangent-modulated exponentially decaying wavelet threshold function is constructed, and, in combination with an improved thresholding criterion, it is applied to suppress the residual noise in the dominant verification components, thereby obtaining the denoised signal. In addition, an ultrasonic sensor verification platform based on a full-scale transformer is developed to verify the reliability of the proposed verification method under noisy conditions. Nonlinear inertia weights and adaptive learning factors are introduced and integrated with permutation entropy. This approach effectively mitigates the susceptibility of conventional methods to local optima under strong noise interference, thereby accelerating the convergence speed. Furthermore, an arctangent-modulated exponential decay wavelet thresholding function is constructed. This function filters out residual noise while maximally preserving the amplitude characteristics critical for sensitivity calculation results. The accuracy of field calibration for partial discharge ultrasonic sensors is significantly enhanced by the proposed method. Consequently, field personnel are enabled to assess the actual operational condition of the sensors.

## 2. On-Site Verification of Transformer Partial Discharge Ultrasonic Sensors and the Influence of Noise

### 2.1. On-Site Verification Method for Ultrasonic Sensors in Transformer Partial Discharge Detection

**Figure 1:**
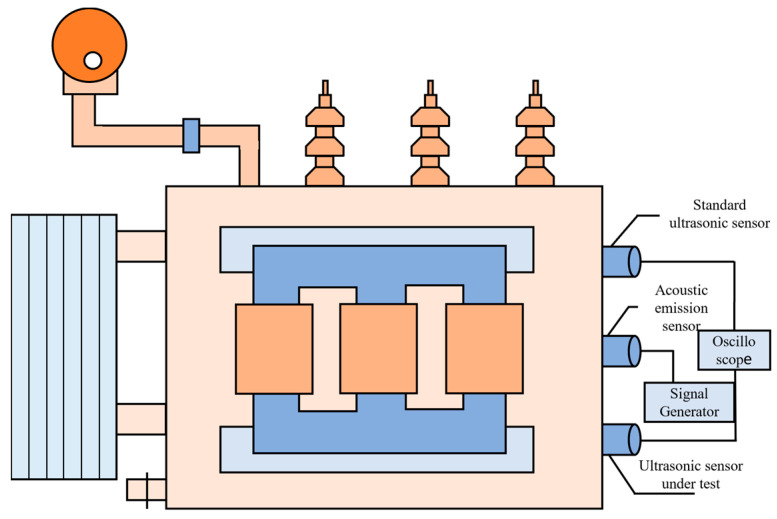
Installation wiring diagram of the ultrasonic sensor field verification method.

**Figure 2:**
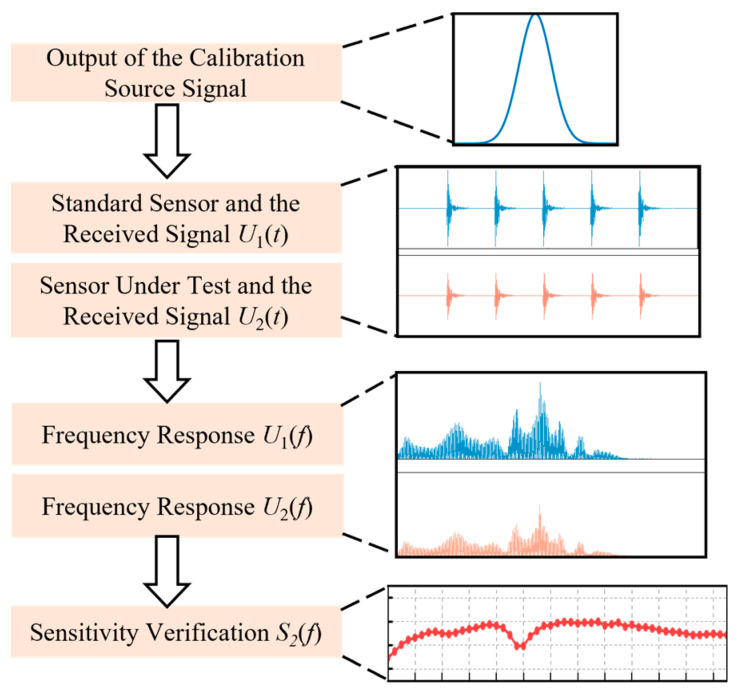
Field verification method flow.

Both GB/T 19801-2005 and Q/GDW 11304.9-2023 specify secondary verification methods and experimental wiring procedures for ultrasonic sensors in laboratory environments [16,17]. In addition to requiring an acoustic emission sensor and a reference sensor, these standards mandate the use of a steel test block with a minimum thickness of 180 mm. However, in field applications, ultrasonic sensors used for transformer partial discharge detection and localization are typically mounted on the transformer tank using silicone adhesive or metal clamps, making them difficult to remove for short periods. Furthermore, the steel verification blocks are bulky and inconvenient to transport. Therefore, based on the constraints of the aforementioned standards and considering the practicality of field verification, this study adopts an on-site verification method for partial discharge ultrasonic sensors directly on the transformer tank. The hardware installation and verification procedures for the on-site method are illustrated in Figure 1 and Figure 2.

Prior to verification, the sensor under testing is used as the reference point, with the acoustic emission sensor and the reference sensor aligned longitudinally. During verification, the acoustic emission sensor outputs a set of Gaussian pulse signals with an amplitude of 10 V, which propagate through the transformer tank. The received signals from the reference ultrasonic sensor and the sensor under testing are denoted as U_1_(*t*) and U_2_(*t*), respectively.

Subsequently, frequency-domain analysis is applied to obtain the frequency responses of the two sensors, *U*_1_(*f*) and *U*_2_(*f*). The sensitivity curve of the sensor under testing is then calculated using Equation (1), where *S*_1_(*f*) represents the sensitivity of the reference sensor, a value typically provided by a third-party testing organization.

(1) $$S_{2}(f)=S_{1}(f)+20\mathrm{lg}\left[\frac{U_{2}(f)}{U_{1}(f)}\right]$$

### 2.2. Analysis of the Effects of Mixed Noise

**Figure 3:**
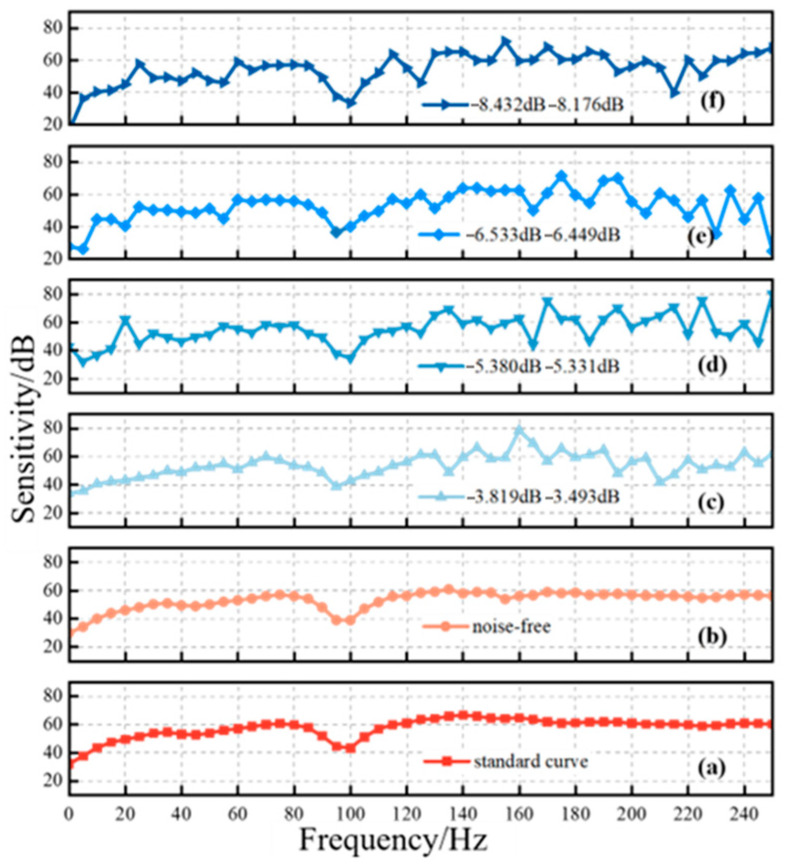
Verification results of ultrasonic sensor sensitivity curve under noise interference. (**a**) Standard sensor sensitivity curve (**b**) Sensitivity curve of the sensor under test under noise influence (**c**) [−5,−6] dB sensitivity curve (**d**) [−6,−7] dB sensitivity curve (**e**) [−8,−9] dB sensitivity curve (**f**) [−3,−4] dB sensitivity curve.

**Figure 4:**
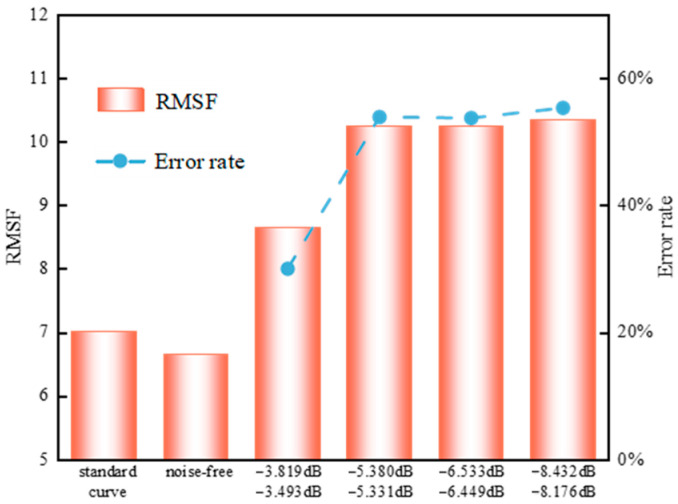
Root mean square fluctuation of sensitivity curve.

Since multiple uncertain interference sources exist in the field, the signals received by sensors are inevitably affected by mixed noise, among which periodic interference and Gaussian noise are the most significant [18,19]. To simulate mixed noise disturbances of different intensities, this study investigates noisy received signals under four signal-to-noise ratio (SNR) levels: [−3 dB, −4 dB], [−5 dB, −6 dB], [−6 dB, −7 dB], and [−8 dB, −9 dB]. To quantitatively characterize the impact of mixed noise on the sensitivity characteristics of sensors, this study, in accordance with existing standards and the noise-related properties of signals, adopts peak sensitivity, mean sensitivity, and the root mean square fluctuation (RMSF) of the sensitivity curve as verification indicators. The sensitivity characteristics of partial discharge ultrasonic sensors were calculated, and the resulting sensitivity curves are shown in Figure 3. Specifically, Figure 3a,b represent the sensitivities of the reference sensor and the sensor under testing without noise interference, while Figure 3c–f correspond to the sensitivity curves of the sensor under testing under different levels of noise interference. As can be observed, the fluctuation of the sensitivity curve increases progressively with a decreasing signal-to-noise ratio (SNR). The RMSF values of each sensitivity curve were further calculated, and the results are presented in Figure 4. It can be seen that noise interference significantly increases this metric, and as the SNR decreases, the RMSF of the curves in Figure 3c–f compared with that of the curve in Figure 3b rises sharply, with the error rate increasing from 30.07% to more than 55.35%.

The mean sensitivity and peak sensitivity of each signal group were calculated, and the results are presented in Table 1. As shown, under mixed noise interference, the variation in peak sensitivity is relatively large, with errors reaching 59.20% or higher. This occurs because as the RMSF of the sensitivity curve increases, data dispersion also rises, causing data points to deviate more easily from the mean. Consequently, the verification result of peak sensitivity exceeds 60 dB (V/(m/s)), indicating that the obtained value is not accurate compared with the true value [17], which may ultimately lead to misjudgment of sensor performance.

## 3. Verification Signal Denoising Method Based on IPSO-Optimized MVMD, EEn, and Improved Wavelet Thresholding

### 3.1. IPSO and Permutation Entropy

The particle swarm optimization (PSO) algorithm performs an optimal value search based on the velocity and position attributes of particles in space, where the position of each particle is regarded as a potential solution [20,21]. During the iterative adjustment process, all particles share their individual best positions as well as the current global best position of the swarm, ultimately converging toward the optimal solution. The velocity and position of particles are updated according to the iterative equations given in (2).

(2) $$\begin{array}{l} v_{i}^{k+1}=\;wv_{i}^{k}+c_{1}r_{1}\;\left(p_{i}^{k}-x_{i}^{k}\right)+c_{2}r_{2}\;\left(p_{g}^{k}-x_{i}^{k}\right)\\ x_{i}^{k+1}=x_{i}^{k}+v_{i}^{k+1} \end{array}$$

In the equations, *v_i_^k^* denotes the velocity of particle *i* in the *k*-th iteration, while *x_i_^k^* represents its position. The parameter *w* is the inertia weight, and *c*_1_ and *c*_2_ are the learning factors. However, the conventional PSO algorithm often suffers from insufficient search capability in the later stages of optimization, which may lead to premature convergence and entrapment in a local optimum. To overcome this limitation and more accurately simulate the actual search process, the inertia weight is adaptively reduced based on the global optimal fitness value. A nonlinear inertia weight particle swarm optimization (IPSO) algorithm is thereby employed to optimize the penalty factor *α* and the number of modes *K* required in MVMD. The proposed calculation formula for the inertia weight *w* is expressed as follows:

(3) $$w=(w_{\max}-w_{\min}){\left(\frac{l}{L}\right)}^{2}\cos(\frac{\pi l}{2L})$$

In this equation, *l* represents the current iteration number, and *L* denotes the maximum number of iterations. The expression illustrates that, during the initial stage of the search, particles are relatively distant from the optimal solution; therefore, a larger inertia weight is adopted to enhance the global search capability. In the later stage, a smaller inertia weight is employed to strengthen local refinement, thereby improving convergence speed.

At the same time, the search capability of IPSO can also be optimized by adjusting the learning factors. The learning factors *c*_1_ and *c*_2_, respectively, determine the weights of a particle’s movement toward its individual best position and the global best position of the swarm. During the early stage of the search, particles must be dispersed throughout the search space to explore local optima; thus, the individual cognitive weight should be relatively large. In the later stage, particles should gradually converge toward the neighborhood of the global optimum, meaning that the global cognitive weight should be emphasized. Therefore, the performance of the algorithm can be improved by gradually decreasing *c*_1_ while increasing *c*_2_. The modified expressions for the learning factors are given as follows:

(4) $$c_{1}=c_{1}^{0}+c_{1}^{0}[{\left(\frac{l}{L}\right)}^{2}-\frac{2l}{L}]$$

(5) $$c_{2}=0.5+c_{2}^{0}(1-e^{-\frac{2l}{L}})$$

Permutation entropy has been widely employed as an indicator for quantifying the randomness of signal sequences and detecting dynamical mutations [22]. For noisy verification signals, the noise components typically exhibit higher permutation entropy values, whereas the effective components of the verification signal, due to their more regular time-domain structure, are associated with lower entropy values. Compared with other complexity measures, permutation entropy is advantageous because of its strong noise robustness and the absence of a requirement for predefined parameters. Therefore, in this study, permutation entropy is adopted as the fitness function. Due to this, IPSO can efficiently identify parameter combinations that minimize the complexity of intrinsic mode functions (IMFs), thereby concentrating the effective signal components within low-entropy modes. The objective function is defined as follows:

(6) $$\mathrm{fitness}(\alpha ,K)=\frac{1}{m}\sum_{i=1}^{m}H_{\mathrm{PE}}(\mathrm{IMF}(i))$$

(7) $$H_{PE}(m)=-\sum_{i=1}^{N-(m-1)\tau }P(i)log_{2}P(i)$$

(8) $$P(i)=\frac{Num(X(i))}{N-(m-1)\lambda }$$

By calculating the minimum value of the above fitness function, the penalty factor and the number of modes required for MVMD can be automatically optimized.

### 3.2. MVMD Optimization and Decomposition Based on IPSO

**Figure 5:**
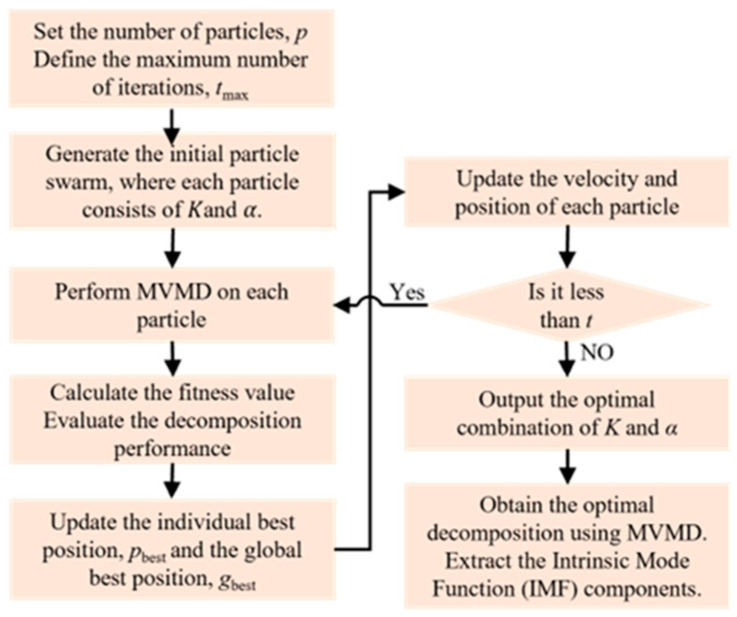
MVMD parameter adaptive optimization process based on IPSO.

**Figure 6:**
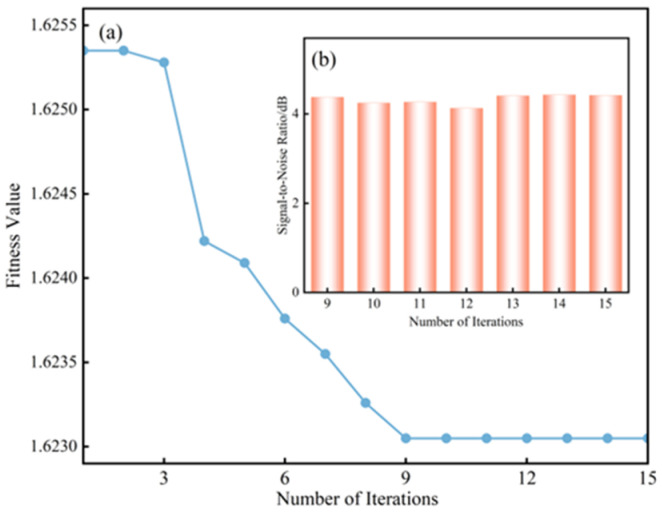
Results of fitness changes during optimization. (**a**) The curve of fitness value changing during the optimization process. (**b**) Optimization effect under different number of iterations.

In the multivariate variational mode decomposition (MVMD) algorithm, the appropriate selection of the number of decomposition modes *K* and the penalty factor *α* directly affects the quality of signal decomposition. An excessively large *K* may lead to signal distortion, whereas an insufficient *K* makes it difficult to effectively separate signal features, resulting in mode mixing. Meanwhile, the choice of the penalty factor *α* must balance the trade-off between frequency band constraint strength and signal fidelity. To address this parameter sensitivity issue, the proposed IPSO method was employed for adaptive parameter optimization. The detailed procedure is illustrated in Figure 5. The search range for *K* was set to [2, 25], while that for *α* was defined as [100, 10,000]. The population size was chosen as 2, and the maximum number of iterations was set to 15. The variation in the fitness values during the optimization process is illustrated in Figure 6a. As shown in the figure, the minimum fitness value was achieved in the ninth iteration. The optimization performance after the ninth iteration was further evaluated by performing MVMD using the corresponding optimized values of *K* and *α*, followed by signal reconstruction to compute the signal-to-noise ratio (SNR). As shown in Figure 6b, the optimization results remained nearly consistent with further iterations, indicating that only nine iterations were sufficient to obtain the optimal parameter combination. These results demonstrate that the proposed IPSO-based method effectively reduces the required number of iterations and accelerates parameter optimization.

**Figure 7:**
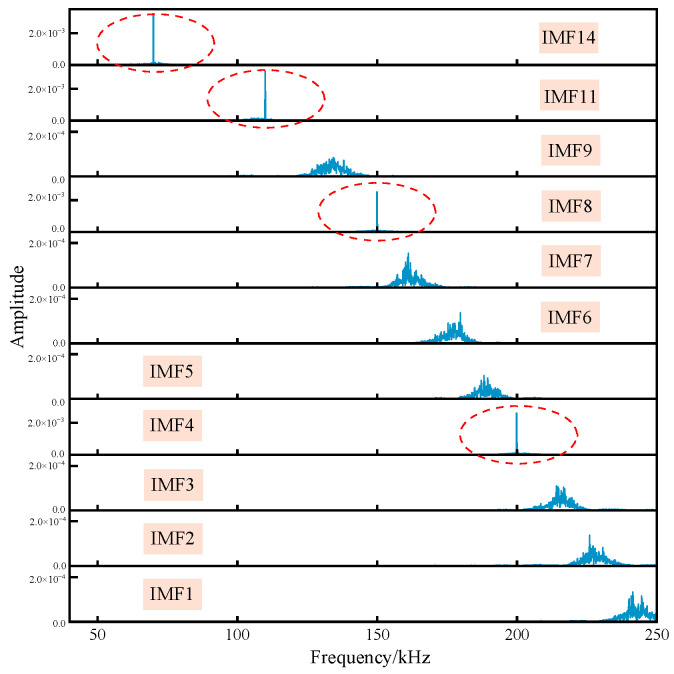
IMF component spectrum dominated by noise component.

The optimal MVMD input parameters obtained from the ninth iteration were *K* = 19 and *α* = 7837. The noisy verification signal was subsequently decomposed using MVMD, and the spectra of the noise-dominated intrinsic mode functions (IMFs) are presented in Figure 7. As shown in the figure, IMF14, IMF11, IMF8, and IMF4 correspond to narrowband noise-dominated components, while the remaining IMFs are primarily dominated by white noise. These results indicate that the IPSO-optimized MVMD method demonstrates excellent modal separation performance, enabling precise separation of narrowband noise, partial white noise, and the respective modal components of the verification signal.

### 3.3. Energy Entropy Criterion

Energy entropy can effectively characterize the attribute features of modal components by quantifying the degree of disorder in the signal distribution within the frequency or time–frequency domain [23]. The energy entropy of each modal component obtained through MVMD is calculated as follows:

(9) $$H_{E}=-\sum_{i=1}^{N}\frac{E_{i}}{\sum_{i=1}^{N}E_{i}}\ln(\frac{E_{i}}{\sum_{i=1}^{N}E_{i}})$$

**Figure 8:**
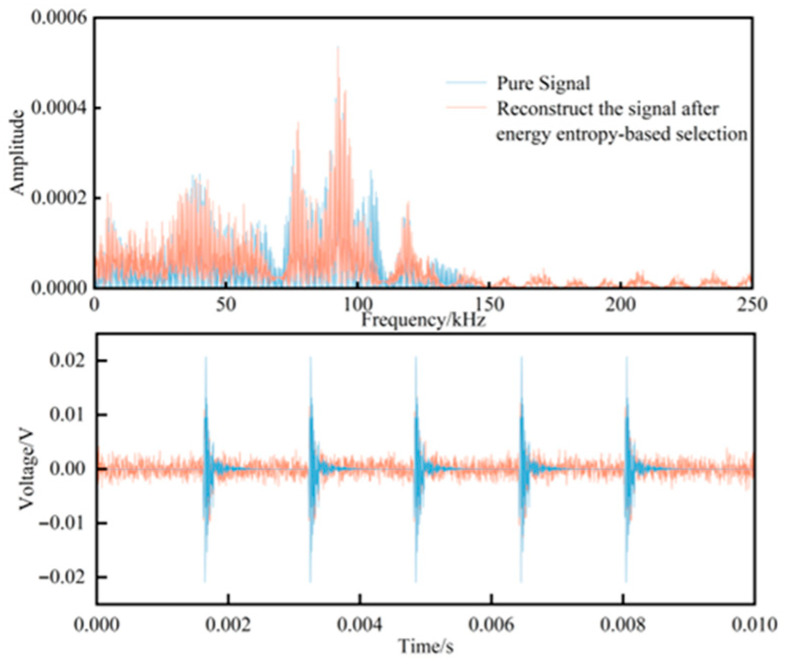
Time–frequency domain diagram of the reconstructed signal after energy entropy screening.

In this formulation, *N* denotes the number of decomposed modes, and *E*_i_ represents the energy of each IMF component. For noise-dominated components, the energy exhibits a broadband and diffuse distribution in the time–frequency domain. Since the energy proportions across different frequency bands tend to become uniform, the corresponding energy entropy values are significantly higher than the reference threshold. In contrast, signal-dominated components exhibit energy concentration within specific frequency bands that reflect intrinsic physical characteristics, thereby forming a distinct local aggregation in the energy probability distribution. As a result, their energy entropy values are reduced by an order of magnitude compared to those of noise components. Based on this property, a threshold can be set such that IMF components with entropy values lower than the threshold are retained as signal-dominated components of the verification signal. After reconstructing the IMF components selected by the energy entropy criterion, the resulting time–frequency distribution is shown in Figure 8. It can be observed that the noise-dominated components are effectively removed, while the signal-dominated components are preserved. However, some residual noise remains, which necessitates further de-noising of the IMF components through the improved wavelet thresholding method.

### 3.4. Wavelet Denoising Based on an Improved Threshold Criterion and Threshold Function

The wavelet thresholding denoising method is capable of progressively refining noisy signals across multiple scales, making it particularly suitable for processing ultrasonic partial discharge verification signals with weak and non-stationary characteristics. The procedure of wavelet thresholding denoising consists of the following steps: first, an appropriate wavelet basis function is selected, and the noisy signal is decomposed in the wavelet domain; second, a suitable thresholding function is applied to suppress or nonlinearly process the noise; finally, the required coefficients of the signal are retained, and the signal is reconstructed to achieve denoising. Therefore, the selection of the wavelet basis and the thresholding strategy are critical factors that determine the effectiveness of the denoising process.

To address the residual noise that remains in the retained IMF components, an improved wavelet thresholding method, incorporating both modified threshold criteria and enhanced thresholding functions, was employed in this study to further extract the characteristic information of the verification signal from each IMF component. The processed components were then reconstructed to obtain the final denoised verification signal. The conventional expressions of the soft and hard thresholding functions are defined as follows:

(10) $$\omega_{s}=\left\{\begin{array}{ll} \;\omega_{j,k} & |\omega_{j,k}|\geq \lambda \\ \;0 & |\omega_{j,k}|<\lambda \end{array}\right.$$

(11) $$\omega_{s}=\left\{\begin{array}{ll} \;\mathrm{sign}(\omega_{j,k})(|\omega_{j,k}|-\lambda ) & |\omega_{j,k}|\geq \lambda \\ \;0 & |\omega_{j,k}|<\lambda \end{array}\right.$$

In these expressions, *λ* denotes the predefined threshold, *ω*_(*j*,*k*)_ represents the unprocessed wavelet coefficients, and *ω_s_* corresponds to the wavelet coefficients after thresholding. Although both thresholding methods exhibit satisfactory denoising performance, their effectiveness is insufficient for signals with low signal-to-noise ratios (SNRs). A comparison of Equations (10) and (11) reveals the underlying reasons: For the hard-threshold function, discontinuity occurs when *ω*_(*j*,*k*)_ = *λ*, which introduces oscillations in the reconstructed verification signal. In contrast, while the soft-threshold function addresses the issue of discontinuity, when the absolute value of *ω*_(*j*,*k*)_ exceeds *λ*, a constant deviation arises between the pre- and post-processed wavelet coefficients, thereby reducing the accuracy of the reconstructed signal.

To overcome the limitations of the aforementioned threshold functions, this study develops a modified wavelet threshold function based on the soft-thresholding expression. Specifically, an arctangent-modulated exponential decay threshold function is constructed, and its mathematical formulation is given as follows:

(12) $$\omega_{s}=\left\{\begin{array}{l} \mathrm{sign}(\omega_{j,k})\left[\left|\omega_{j,k}\right|-\lambda e^{-({\left(\frac{\left|\omega_{j,k}\right|}{\lambda }\right)}^{2}-1)\arctan(\frac{\left|\omega_{j,k}\right|}{\lambda }-1)}\right],\left|\omega_{j,k}\right|\geq \lambda \\ 0,\left|\omega_{j,k}\right|<\lambda \end{array}\right.$$

**Figure 9:**
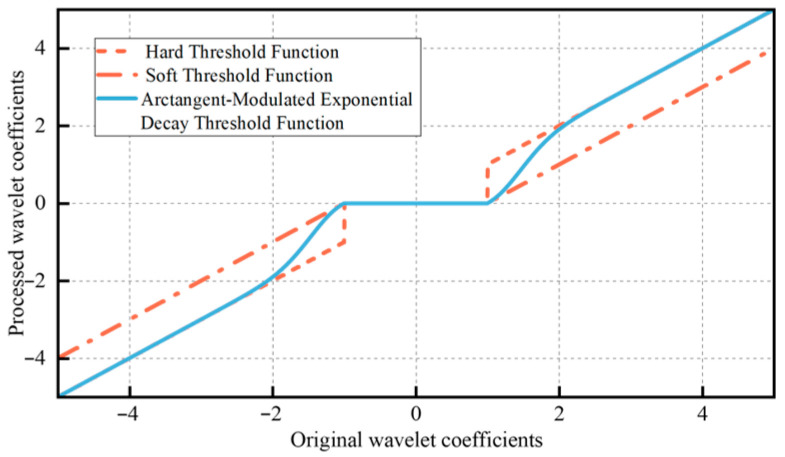
Improved threshold function image.

As illustrated in Figure 9, the proposed threshold function exhibits a smoother transition compared with both the soft and hard threshold functions. In addition, the traditional threshold value is further improved in this study, with its formulation expressed as follows:

(13) $$\lambda =\frac{1.9\sigma^{2}}{\sqrt{\sigma_{c}^{2}-\sigma^{2}}}$$

**Figure 10:**
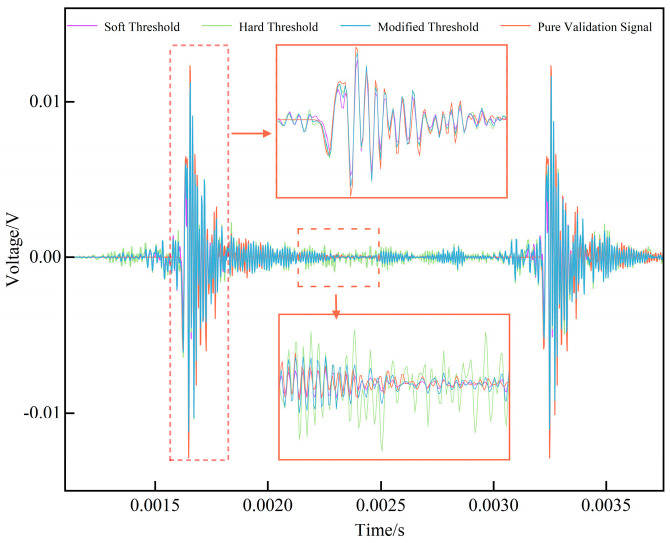
Comparison of results under different threshold functions.

In this expression, *σ*_c_ denotes the standard deviation of the noisy wavelet coefficients, while *σ* represents the noise standard deviation estimated in the previous step. The proposed improved wavelet thresholding method was applied for signal denoising and the outcomes were compared with results obtained using different threshold functions, as illustrated in Figure 10. The results demonstrate that denoising with the hard threshold function leaves a significant amount of residual noise during the stationary stage of the signal, while denoising with the soft threshold function leads to a considerable reduction in amplitude at the signal peaks. Both approaches therefore yield suboptimal performance. In contrast, the signal denoised using the improved thresholding criterion and threshold function exhibits a waveform more closely aligned with the original verification signal, thereby achieving superior denoising performance.

## 4. Simulation and Analysis

### 4.1. Noise Suppression of the Verification Signal

**Figure 11:**
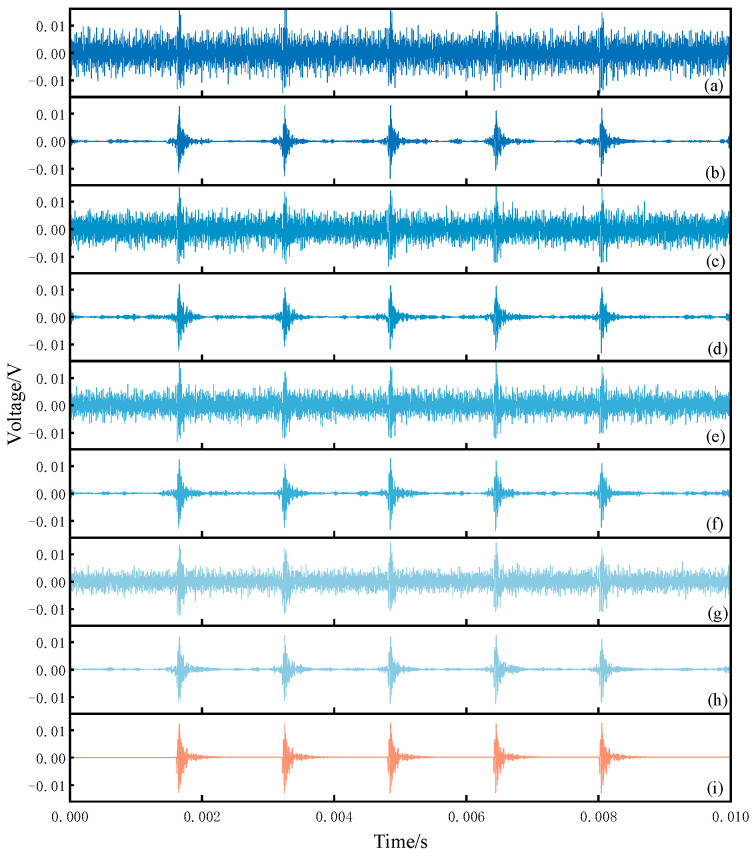
Time domain comparison of analog signals before and after denoising. (**a**) [−8,−9]dB Noisy signal (**b**) [−8,−9]dB Denoising signal (**c**) [−6,−7]dB Noisy signal (**d**) [−6,−7]dB Denoising signal (**e**) [−5,−6]dB Noisy signal (**f**) [−5,−6]dB Denoising signal (**g**) [−3,−4]dB Noisy signal (**h**) [−3,−4]dB Denoising signal (**i**) Noisy-free signal.

The time-domain waveforms of verification signals under mixed noise with different intensities are shown in Figure 11a,c,e,g, corresponding to the four signal-to-noise ratio (SNR) levels defined in Section 2.2. The noisy signals from both the standard sensor and the sensor under verification were obtained using simulation. Prior to decomposition, the multivariate variational mode decomposition (MVMD) parameters, namely the number of modes *K* and the penalty factor *α*, were optimized using the improved particle swarm optimization (IPSO) algorithm. For the four noisy signals, the optimal number of modes was determined as *K* = 19, while the corresponding penalty factor *α* was found to lie within the range of [7000–8500]. The noisy verification signal was decomposed into 19 intrinsic mode functions (IMFs). To extract the IMFs dominated by the verification signal, the energy entropy of each component was calculated, and components with entropy values exceeding the threshold of 0.91 were removed. These discarded components correspond to IMFs dominated by narrowband noise and portions of broadband noise. To further suppress the residual broadband noise present in the extracted IMFs, an improved wavelet thresholding method was applied to extract the characteristic verification signal from the IMFs, which were then reconstructed to obtain the final denoised verification signal.

During the verification process, both the standard sensor and the sensor under testing were exposed to the same environment and were therefore affected by noise. To ensure high verification accuracy, the received signals of both types of sensors had to undergo denoising. Taking the verification signal received by the sensor under testing as an example, the denoising results under different levels of mixed noise are shown in Figure 11b,d,f,h, while Figure 11i presents the noise-free reference signal received by the sensor under testing. A comparison between the denoised signals and the reference signal demonstrates that the proposed method achieved effective noise suppression across different signal-to-noise ratio (SNR) conditions. To quantitatively evaluate the denoising performance, the SNR and normalized cross-correlation (NCC) were employed as evaluation metrics [24], and the corresponding results are presented in Table 2. For signals with an SNR lower than –5 dB, representing cases with strong noise interference, the proposed method was able to maintain the SNR of the denoised signals above 8 dB. In cases of weaker noise interference, the method achieved an SNR exceeding 10 dB.

**Figure 12:**
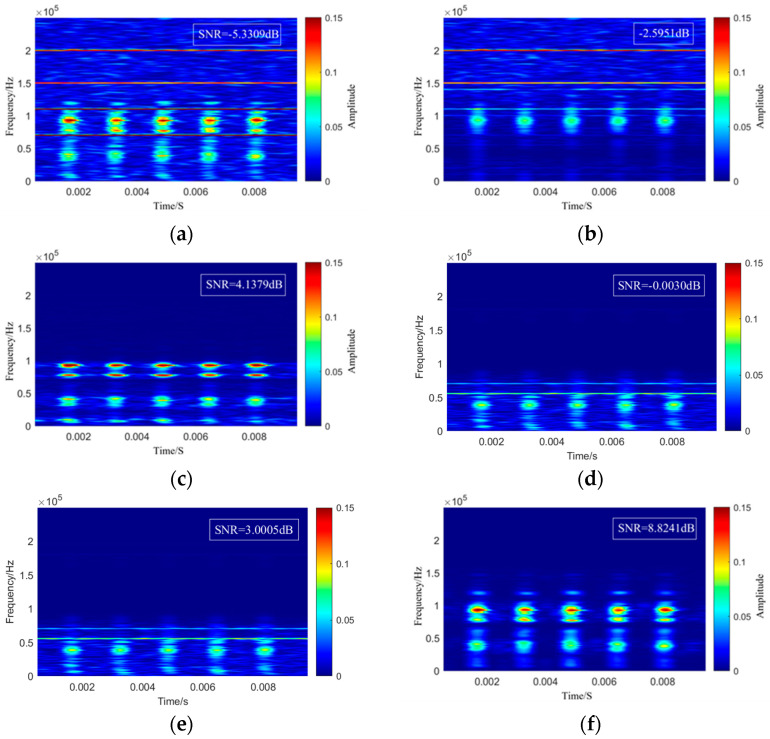
Comparison of time–frequency graphs of denoising results under different denoising methods. (**a**) Noisy signal of the sensor under testing. (**b**) Denoising result using CEEMDAN–wavelet thresholding. (**c**) Denoising result using VMD–ICA. (**d**) Denoising result using VMD. (**e**) Denoising result using the conventional wavelet threshold. (**f**) Denoising result using the proposed method.

To further verify the reliability and superiority of the proposed method for noise suppression in ultrasonic sensor verification signals, the noisy signal shown in Figure 11e was processed using the CEEMDAN–wavelet thresholding method, the VMD-ICA method, the VMD method, and the conventional wavelet threshold method. The denoised results obtained from these approaches were then compared with those produced by the proposed method. The signal-to-noise ratio (SNR), normalized cross-correlation (NCC), and root mean square error (RMSE) were calculated as quantitative evaluation metrics. The corresponding time–frequency comparison results are presented in Figure 12. As shown in the comparison results in Figure 12 and the quantitative evaluation metrics in Table 3, the CEEMDAN–wavelet thresholding method failed to completely suppress narrowband noise and caused distortion in the primary components of the original verification signal. This is because the frequencies of the narrowband interference were very close to certain inherent frequencies of the original signal, leading both the noise and signal to be decomposed into the same IMF component, which prevented the subsequent wavelet thresholding method from effectively separating them. In the denoising results obtained by the VMD-ICA method, a considerable amount of residual white noise remained within the frequency range of the verification signal. This occurred because the IMF components retained after VMD still contained white noise. When such residual noise approximates a Gaussian distribution, its presence within the IMF components becomes difficult to capture using the non-Gaussianity criterion of ICA. Furthermore, once the residual noise is mixed with the verification signal in the IMF components, the relationship is no longer a simple linear superposition, which violates the statistical independence assumption required by ICA, thereby limiting its ability to effectively distinguish noise components. While the VMD method effectively suppressed periodic interference, significant residual Gaussian noise remained in the denoised signal. Consequently, although the NCC showed improvement, the SNR remained at a relatively low level. This limitation is attributed to the fact that, while VMD is highly sensitive to periodic interference, it struggles to distinguish the target signal from noise when their energy profiles and spectral characteristics overlap. Conversely, conventional wavelet methods effectively suppress Gaussian noise but fail to adequately eliminate periodic interference, resulting in severe waveform distortion. As indicated by the quantitative data in Table 3, both the SNR and NCC for these methods were low. This performance deficit arose because conventional wavelet methods are primarily suited for scenarios involving broadband noise and are highly dependent on the precise selection of thresholding functions. In contrast, the proposed method successfully suppressed different types of noise, achieving the highest improvement in SNR, an NCC value closest to 1 (0.9324), and an RMSE value closest to 0 (0.00048). These results demonstrate the superior performance of the proposed approach.

### 4.2. Analysis of Sensitivity Verification Results Based on Noise Suppression

**Figure 13:**
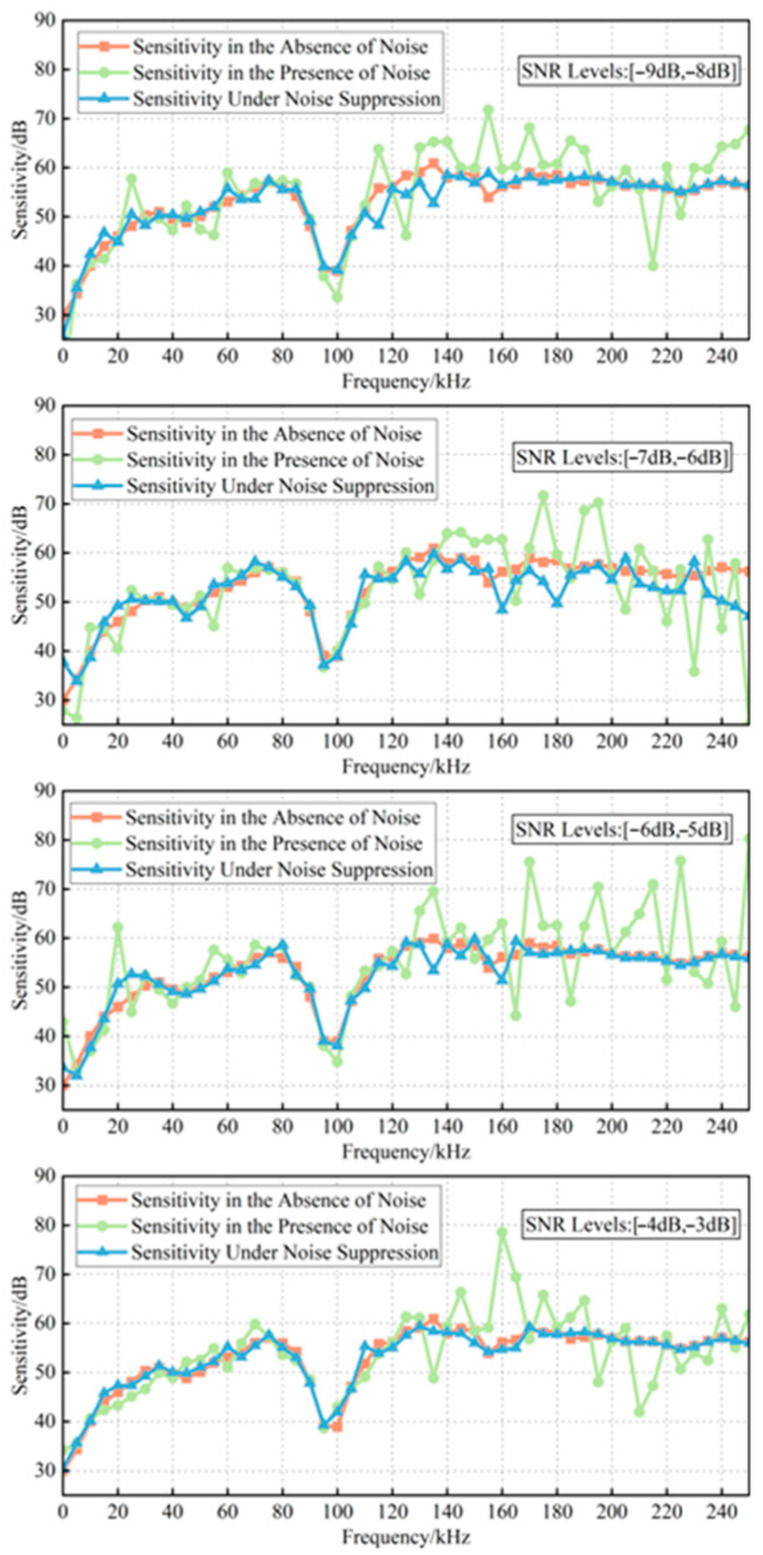
Comparison of sensitivity curves before and after noise suppression.

After confirming that the proposed method demonstrated strong noise suppression performance for the signals themselves, its impact on ultrasonic sensor sensitivity verification was further examined. The noisy verification signals from both the standard sensor and the sensor under testing were processed using the proposed denoising approach. Following the verification procedure described in Section 2.1, the denoised signals were then applied to calculate the sensitivity of the sensor under testing. The corresponding results are shown in Figure 13. Compared with the sensitivity curve obtained before denoising, the sensitivity curve after noise suppression remains highly consistent with the original sensitivity curve of the sensor under testing, exhibiting good agreement without significant fluctuations. The calculated sensitivity verification results are summarized in Table 4. After noise suppression, the errors of the mean sensitivity and peak sensitivity were maintained within 1.108 V/m/s and 0.727 V/m/s, respectively, while the RMSF error remained stable within 10%. When the performance of the sensor under testing degraded, its peak sensitivity dropped below the standard threshold of not less than 60 dB specified in Q/GDW 11304.9-2023. Without noise suppression, the presence of noise could cause this indicator to appear greater than 60 dB; however, the proposed method effectively restores the true value of the sensor’s performance index, thereby preventing potential misjudgments by field personnel. Overall, these findings demonstrate that the proposed noise suppression method significantly enhances the accuracy and reliability of ultra-sonic sensor sensitivity verification.

## 5. Construction of the Verification Platform and Analysis of Measured Verification Signals

**Figure 14:**
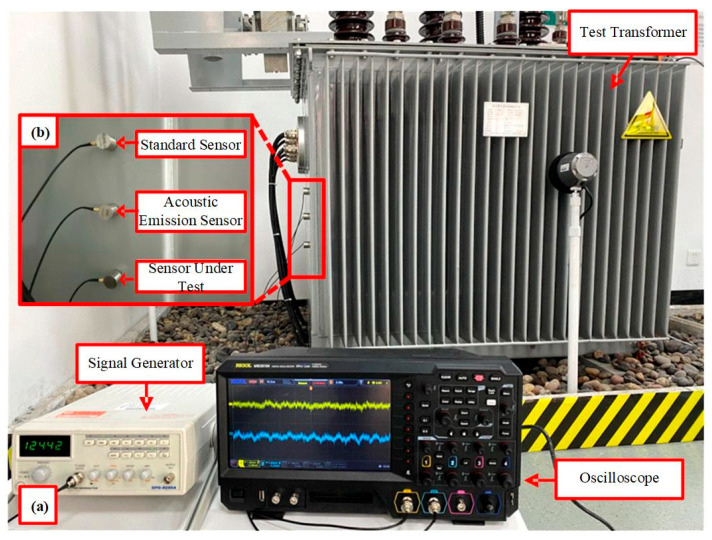
(**a**) Transformer partial discharge ultrasonic sensor verification platform. (**b**) Enlarged view of sensor arrangement.

To further validate the effectiveness of the proposed method for on-site verification of ultrasonic sensors, a partial discharge ultrasonic sensor verification platform was constructed based on a 35 kV transformer. The proposed denoising approach was applied to the measured verification signals, and the sensitivity verification results were subsequently analyzed. As shown in Figure 14, and according to the sensor and equipment installation and wiring diagram presented in Section 2.1, the acoustic emission sensor, the standard sensor, and the sensor under verification were first affixed to the surface of the transformer tank with an interval of 100 mm between them, arranged in a vertical configuration. To facilitate validation, another standard sensor was used in place of the sensor under verification, for which the sensitivity curve under noise-free conditions was provided by the manufacturer. Subsequently, the signal generator was connected to the acoustic emission sensor via a BNC cable, while the oscilloscope was connected to both the standard sensor and the sensor under verification using BNC cables. Finally, the signal generator and oscilloscope were powered on, completing the setup of the verification platform.

**Figure 15:**
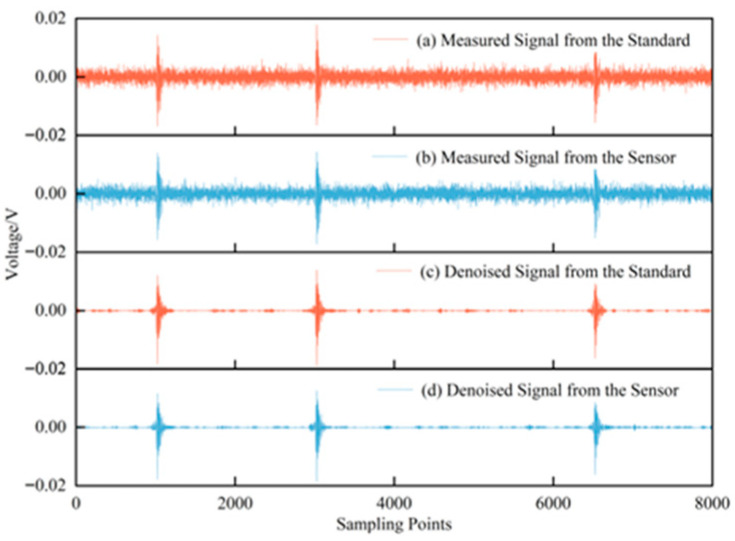
Time-domain comparison of measured signals before and after denoising.

**Figure 16:**
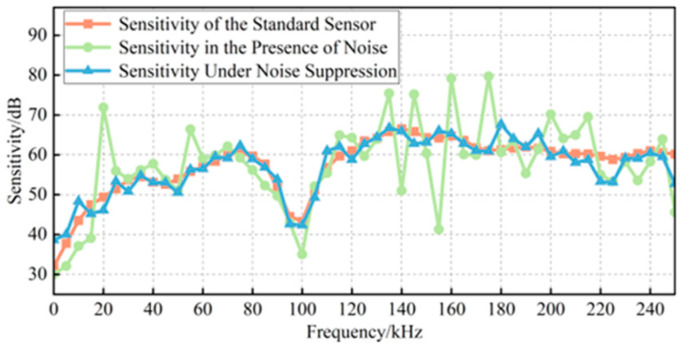
Comparison of experimental sensitivity curves.

The signal generator output a set of steep pulse signals to excite the acoustic emission sensor, which emitted ultrasonic signals. The signals received by the standard sensor and the sensor under verification are shown in Figure 15a,b, while the results after denoising with the proposed method are presented in Figure 15c,d. As illustrated, the proposed method effectively suppressed on-site noise to the greatest extent while preserving the essential characteristics of the original signals. To more directly demonstrate the impact of noise suppression on sensor verification, the sensitivity curves and verification indices before and after denoising were calculated, as shown in Figure 16. Using the proposed denoising approach, the RMSF of the sensitivity curve was reduced from 10.9857 to 7.1153, and the curve closely aligned with the true sensitivity curve provided by the manufacturer. Furthermore, the error rates of both the mean sensitivity and peak sensitivity were maintained below 4%. Therefore, the proposed method plays a significant role in enhancing the accuracy of on-site verification of ultrasonic sensors.

## 6. Conclusions

In this study, a noise suppression method for on-site ultrasonic sensor verification was proposed, which integrates IPSO-optimized MVMD, energy entropy (EEn), and an improved wavelet threshold. Both simulation and experimental analyses demonstrated that the proposed method significantly enhances the accuracy of ultrasonic sensor field verification. The main conclusions are as follows:

(1) By improving the inertia weight and learning factors, the IPSO algorithm is optimized, with permutation entropy employed as the fitness function to achieve automatic parameter optimization in MVMD. This approach effectively reduces the number of iterations and overcomes the difficulty of modal decomposition caused by spectral aliasing due to the coexistence of verification signals and noise under practical operating conditions.
(2) Both the wavelet threshold and the threshold function were improved by constructing an arctangent-modulated exponential decay wavelet threshold function. This enhancement strengthened the fitting performance of the verification signal and maximized the suppression of residual noise within the modal components.
(3) A field verification platform for transformer partial discharge ultrasonic sensors was constructed, and the effectiveness of the proposed method in sensor field verification was demonstrated. Future work will focus on advancing the integration and miniaturization of verification equipment.

## Figures and Tables

**Table 1:** Average sensitivity and peak sensitivity of sensor to be verified under noise interference.

Sensor Type	SNR/dB	Mean Sensitivity/(V/m/s)	Peak Sensitivity/(V/m/s)
Standard Sensor	Noise-Free	57.285	66.548
Sensor Under Testing (SUT)	Noise-Free	52.868	59.112
	−3.819/−3.493	53.523	94.109
	−5.380/−5.331	55.329	95.247
	−6.533/−6.449	52.436	102.195
	−8.432/−8.176	54.320	107.434

**Table 2:** Quantitative indicators of denoising results under different noise intensities.

		[−3, −4] dB	[−5, −6] dB	[−6, −7] dB	[−8, −9] dB
**Standard sensor**	**SNR** /dB	10.3221	9.1813	9.2865	8.6368
	NCC	0.9531	0.9379	0.9402	0.9291
**Sensor under testing**	**SNR** /dB	10.3433	8.8241	8.3728	8.2151
	**NCC**	0.9528	0.9324	0.9243	0.9216

**Table 3:** Evaluation indicators of denoising effects of different denoising methods.

Denoising Method	SNR/dB	NCC	RMSE
/	−5.3309	0.4664	0.0024
CEEMDAN–wavelet thresholding	−2.5951	0.2905	0.0018
VMD-ICA	4.1379	0.8007	0.00083
VMD	3.1035	0.8036	0.00088
conventional wavelet threshold	−0.0030	0.3666	0.0013
proposed method	8.8241	0.9324	0.00048

**Table 4:** Quantitative indicators for sensor sensitivity verification under noise suppression.

Signal-to-Noise Ratio/dB	Mean Sensitivity/(V/m/s)	Peak Sensitivity/(V/m/s)	RMSF
**Noise-free**	52.868	59.112	6.6161
[−4 dB, −3 dB]	52.883	59.401	6.2481
[−6 dB, −5 dB]	52.634	59.817	6.4634
[−7 dB, −6 dB]	51.760	59.839	5.9809
[−9 dB, −8 dB]	52.635	58.782	6.6576

## Data Availability

The original contributions presented in the study are included in the article; further inquiries can be directed to the corresponding author.
